# Muscadine grape skin extract reverts snail-mediated epithelial mesenchymal transition via superoxide species in human prostate cancer cells

**DOI:** 10.1186/1472-6882-14-97

**Published:** 2014-03-12

**Authors:** Liza J Burton, Petrina Barnett, Basil Smith, Rebecca S Arnold, Tamaro Hudson, Kousik Kundu, Niren Murthy, Valerie A Odero-Marah

**Affiliations:** 1 Center for Cancer Research and Therapeutic Development, Department of Biological Sciences, Clark Atlanta University, 223 James P Brawley Dr SW, 30314 Atlanta, GA, USA; 2 Department of Pathology, Emory University School of Medicine, Washington, DC 20060, USA; 3 Department of Medicine, Howard University, Washington, DC 20060, USA; 4 Department of Biomedical Engineering, Georgia Institute of Technology, Atlanta, Georgia

**Keywords:** Muscadine grape skin extract, Snail, EMT, Reactive oxygen species, Superoxide, Prostate cancer

## Abstract

**Background:**

Snail transcription factor can induce epithelial-mesenchymal transition (EMT), associated with decreased cell adhesion-associated molecules like E-cadherin, increased mesenchymal markers like vimentin, leading to increased motility, invasion and metastasis. Muscadine grape skin extract (MSKE) has been shown to inhibit prostate cancer cell growth and induce apoptosis without affecting normal prostate epithelial cells. We investigated novel molecular mechanisms by which Snail promotes EMT in prostate cancer cells via *Reactive Oxygen Species* (ROS) and whether it can be antagonized by MSKE.

**Methods:**

ARCaP and LNCaP cells overexpressing Snail were utilized to examine levels of reactive oxygen species (ROS), specifically, superoxide, *in vitro* using Dihydroethidium (DHE) or HydroCy3 dyes. Mitosox staining was performed to determine whether the source of ROS was mitochondrial in origin. We also investigated the effect of Muscadine grape skin extract (MSKE) on EMT marker expression by western blot analysis. Migration and cell viability using MTS proliferation assay was performed following MSKE treatments.

**Results:**

Snail overexpression in ARCaP and LNCaP cells was associated with increased concentration of mitochondrial superoxide, *in vitro*. Interestingly, MSKE decreased superoxide levels in ARCaP and LNCaP cells. Additionally, MSKE and *Superoxide Dismutase* (SOD) reverted EMT as evidenced by decreased vimentin levels and re-induction of E-cadherin expression in ARCaP-Snail cells after 3 days, concomitant with reduced cell migration. MSKE also decreased Stat-3 activity in ARCaP-Snail cells.

**Conclusions:**

This study shows that superoxide species may play a role in Snail transcription factor-mediated EMT. Therefore, therapeutic targeting of Snail with various antioxidants such as MSKE may prove beneficial in abrogating EMT and ROS-mediated tumor progression in human prostate cancer.

## Background

The muscadine grape possesses one of the highest antioxidant levels among fruits; yet, the effect of this fruit on mammalian metabolic systems has not received significant attention [1]. Muscadine Grape Skin Extract (MSKE) derived from the muscadine grape (*Vitis rotundifolia*) differs from the more common red grapes used to produce red wines, which normally contain resveratrol. Based on the skin color, muscadine varieties are referred respectively as bronze and purple compared to white and red for all other grapes [2]. Muscadine grapes are native to Southeastern United States and can be found growing wild from Delaware to the Gulf of Mexico and westward from Missouri to Texas [3]. In a study done by Hudson et al., it was determined that the major phytochemical constitute of MSKE is anthocyanin 3,5-diglucosides which is different from resveratrol [4]. The phenolic structure of anthocyanins is responsible for their antioxidant activity (ability to scavenge ROS) [5]. Reactive Oxygen Species (ROS) that includes superoxide and hydrogen peroxide species has been associated with several disease states including cancer [6]. For example, prostate cancer is associated with aberrant changes in ROS and higher oxidative stress has been found in benign epithelium of prostate cancer patients as compared to men without the disease [7,8]. Increased hydrogen peroxide levels has been reported in exhaled breath condensate in patients with localized breast malignancy, which correlated with clinical severity [9]. Additionally, increased superoxide species were detected in breast and colon carcinoma [10]. With regards to ROS and EMT; *Transforming Growth Factor*-*Beta* (TGF-β-mediated EMT involved increased hydrogen peroxide and *Mitogen*-*activated protein kinase*/*Extracelluar signal reduced kinase* (MAPK/ERK) signaling in proximal tubular epithelial cells [11], while *Matrix Metalloproteinase*-*3* (MMP-3) mediated EMT in mammary epithelial cells involved increase in ROS and Snail [12]. Overexpression of Snail in ARCaP prostate cancer cells has been shown to induce EMT and ROS (hydrogen peroxide and superoxide), possibly by regulating oxidative stress-responsive genes [13]. Some of the transcription factors known to be involved in immediate early gene expression are also regulated by ROS.

Snail transcription factor, a zinc finger protein, can induce EMT which is associated with repression of E-cadherin and induction of vimentin expression and leads to increased cell invasion and migration [14]. Snail has been shown to be associated with increased tumor motility and invasion by induction of epithelial-mesenchymal transition (EMT) [15]. Snail represses E-cadherin transcription *in vitro* and *in vivo* by binding to 5’-CACCTG-3’ sequence in the E-cadherin promoter [16]. Epithelial cells that ectopically express Snail adopt a fibroblastic phenotype and acquire tumorigenic and invasive properties [17]. Previous reports have shown that ARCaP and LNCaP prostate cancer cells stably transfected with Snail displayed decreased adhesion and increased cell migration [15]. It is also reported that Snail confers resistance to cell death [18], which provides a selective advantage for tumors that become malignant.

Signal transducers and activators of transcription (STAT) are proteins that regulate gene expression by affecting transcription. Activation of the *Janus Kinase*/*Signal transducers and activators of transcription* (JAK/STAT) pathway has also been observed in response to generation of intracellular ROS and exogenous hydrogen peroxide (H_2_O_2_) STATs have been implicated in cell growth and survival during oncogenesis. STAT3 has been shown to regulate transcription factors such as twist and the Snail family that are able regulate E-cadherin expression during EMT. Using the ARCaP model, *Zhau et al*. [19] demonstrated a link between LIV-1, a downstream target of STAT3 and EMT. The authors concluded that signaling through STAT3-Snail-LIV-1 resulted in an increased expression of receptor activator of nuclear factor kB (NF-kB) ligand, which facilitates bone metastasis during prostate cancer progression Constitutive activation of STAT-3 has been observed in many human tumors including prostate [20]. The present study has gone further to elucidate specifically the origin and role of superoxide species in the EMT process and whether it can be antagonized by MSKE. We have previously established an ARCaP and LNCaP human prostate cancer EMT cell model by overexpression of Snail transcription factor [21,22]. Utilizing these models, we report here that Snail-mediated EMT is partly regulated by mitochondrial superoxide signaling in prostate cancer cells. Additionally, the antioxidants MSKE and SOD inhibit Snail-mediated superoxide, and reverts EMT. Finally, we show that MSKE may antagonize Snail-mediated signaling by inhibiting the JAK/STAT pathway.

## Methods

### Reagents and antibodies

RPMI 1640 medium (1X with L-glutamine and without L-glutamine and phenol red medium) and penicillin-streptomycin were from Mediatech (Manassas, VA). Protease inhibitor cocktail was from Roche Molecular Biochemicals, Indianapolis, IN. Mouse monoclonal anti-human E-cadherin antibody and rat tail collagen were from BD Transduction Laboratories, Lexington, KY. Mouse monoclonal anti-human vimentin antibody and mouse monoclonal anti-human Stat3 was from Santa Cruz Biotechnology, Santa Cruz, CA. Rabbit monoclonal anti-phospho-Stat3 were from EMD Millipore (Billerica, MA). Phorbol 12-myristate 13-acetate (PMA), and mouse monoclonal anti-human actin antibody were from Sigma-Aldrich, Inc., St Louis, MO. Geneticin (G418) and superoxide dismutase (SOD) were from EMD Corp BioScience (Brookfield, WI). MSKE was kindly provided by Dr Tamaro Hudson, Howard University, Washington, DC, and the preparation of the extract has been previously described [4]. Rat monoclonal anti-human Snail antibody and Horseradish perioxidase (HRP)-conjugated goat anti-rat antibody were from Cell Signaling Technology, Inc., Danvers, MA. HRP-conjugated sheep anti-mouse and the Enhanced chemiluminescence (ECL) detection reagent were purchased from Amersham Biosciences, Buckingham, England. Fetal bovine serum (FBS) and Charcoal/dextran treated FBS (DCC-FBS) were from Hyclone, South Logan, UT. Dihydroethidium bromide (DHE) and Mitosox staining kit were obtained from Invitrogen, Carlsbad, CA. Hydro-Cy3 dye was kindly provided by Dr Niren Murthy, Georgia Institute of Technology, Atlanta, GA. The Snail cDNA construct was kindly provided by Dr Mien-Chie Hung, University of Texas, Houston, TX.

### Cell lines and culture

LNCaP human prostate cancer cells were obtained from ATCC (Manassas, VA). LNCaP cells stably transfected with constitutively active Snail cDNA has been described previously [22]. Cells were grown in RPMI supplemented with 10% fetal bovine serum and 1X penicillin-streptomycin, at 37°C with 5% CO_2_ in a humidified incubator.

### Transfection assay

Stable transfection of Snail cDNA was performed in epithelial ARCaPE cells [23,24] utilizing Lipofectamine 2000 (Invitrogen) as previously described [21] to generate ARCaP-Neo, ARCaP-Snail low, and ARCaP-Snail high clones.

### Western blot analysis

Western blot was performed as described previously [21]. The membranes were stripped using stripping buffer (Pierce Biotechnology, Inc., Rockford, IL) prior to re-probing with a different antibody. For treatments, 70% confluent cells were serum-starved in phenol red-free serum-free RPMI containing penicillin/streptomycin for 24 h prior to treatment with MSKE or SOD in phenol-free serum-free RPMI containing 5% FBS DCC-FBS for 3 days.

### In vitro measurement of superoxide with DHE

For *in vitro* experiments, 70% confluent cells were washed with PBS followed by trypsin digestion. Cells were pelleted at 300 g for 2 min, the supernatant removed and the cells resuspended in 500 μL of HANKS with 5% FBS. 10 μM DHE (to detect superoxide) was added to cells, followed by incubation for 30 min while gently rocking in the dark. 20,000 cells were gated and analyzed by Fluorescence Activated Cell Sorting (FACS).

### In vitro measurement of superoxide with HydroCy3

20,000 cells were plated in RPMI without antibiotics in a 6-well plate. The cells were then placed overnight in 37°C with 5% CO_2_ in a humidified incubator. The next day cells were serum starved in RPMI without L-glutamine and phenol red for three hours followed by replacement of media with 90 μL PBS/HEPES buffer plus 10 μL of 25 μM Hydro-Cy3 for 15 min at 37°C, and subsequent imaging with a fluorescence microscope. To measure superoxide in cell lysate, 100 μl whole cell lysates prepared from untreated or treated cells was mixed with 90 μL HEPES/PBS buffer and 10 μL of 25 μM of HydroCy3 for 1 h followed by OD measurement at 530/590 nm. Protein concentration was assayed with BCA reagent in whole cell lysates to be used to normalize OD readings.

### Mitosox staining

5,000 cells were plated overnight in RPMI supplemented with 10% fetal bovine serum and 1X penicillin-streptomycin in 16-well chamber slides. The MitoSOX staining was performed as per manufacturer protocol. Briefly, 1 mL of 5 μM MitoSOX reagent was added to the cells, covered with foil and placed at 37°C with 5% CO_2_ in a humidified incubator for 10 minutes. The cells were then washed three times with warm HBS/CA/Mg buffer. Cells were counter-stained with DAPI to view the nucleus and images taken with a fluorescence microscope.

### In vitro cell migration assay

We utilized Costar 24-well plates containing a polycarbonate filter insert with an 8-μ pore size, coated with collagen I. Following treatment with MSKE, N-acetylcysteine (NAC), or SOD for 3 days as described above, cells were trypsinized and 50,000 cells were plated in the upper chamber of the insert containing 0.1% fetal bovine serum (FBS) while the lower chamber contained 10% FBS. Five h later (for ARCaP) or 24 h later (for LNCaP), cells that had migrated to the bottom of the insert were fixed, stained, and counted to obtain the relative migration.

### Statistical analysis

Statistical analysis was performed using ANOVA and Turkey’s Multiple Comparison Test as Post-Hoc test from Graph Pad Prizm3 software; *p < 0.05 was considered significant. Multiple experiments were performed in replicates of 3.

## Results

### Mitochondria is the Source of Superoxide Species in ARCaP Cells Transfected with Snail

**Figure 1:**
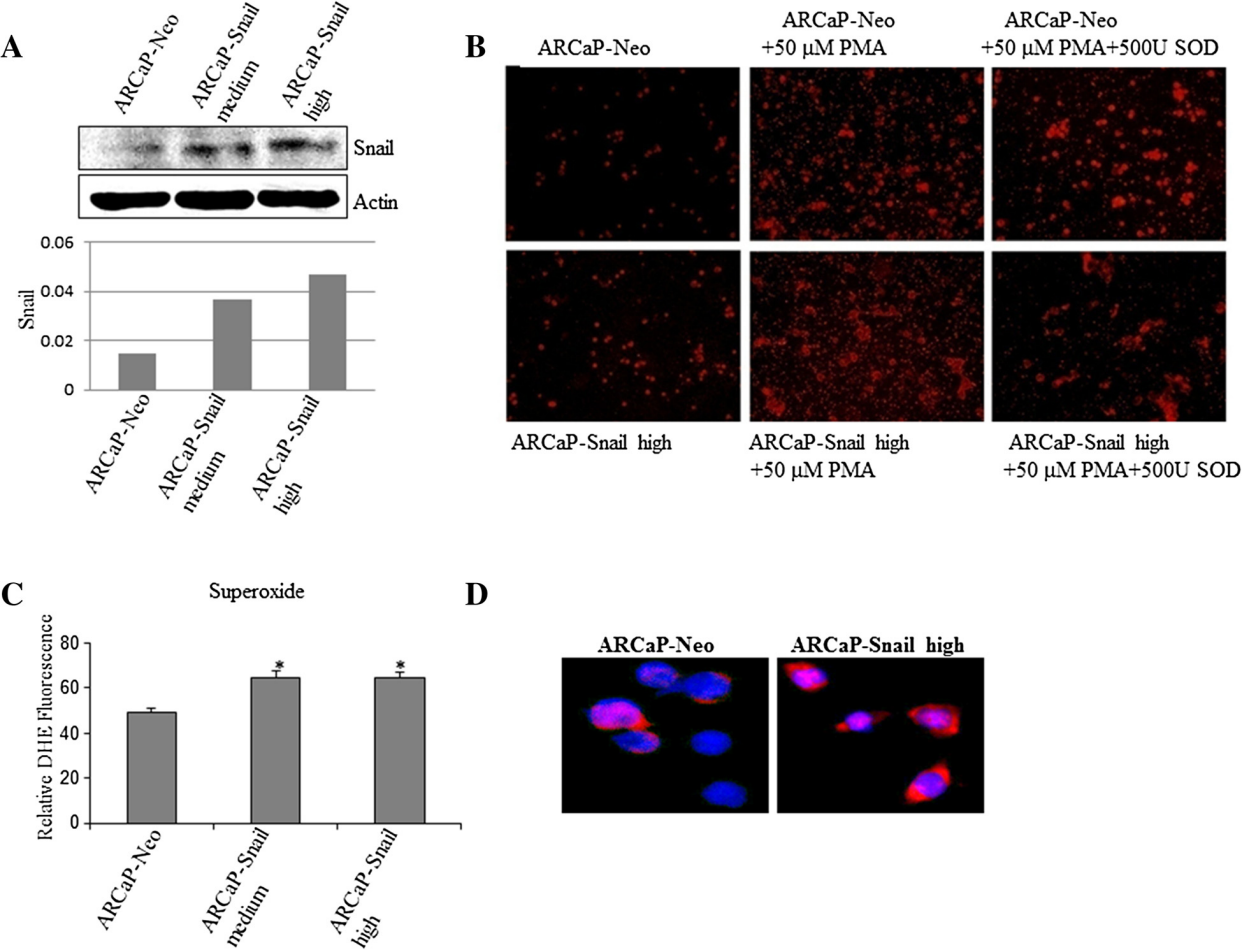
**Snail is overexpressed in ARCaP prostate cancer cell line is associated with increased superoxide *in vitro*. (A)** ARCaPE cells transfected stably with empty vector (ARCaP-NeoL) or Snail cDNA (ARCaP-Snail med, -Snail high) were utilized to analyze Snail levels by Western blot analysis. Quantification of the Western blot results by densitometry with normalization to actin levels was done using the Quantity One quantification software (BioRad). **(B)** 20,000 ARCaP Neo control or Snail-transfected clones were plated in 6-well plates overnight. The next day the cells were serum starved for 3 h, then treated with 50 μM PMA plus or minus 500 U/ml SOD for live cells. Subsequently, the media was replaced with HEPES/PBS buffer containing 25 μM of Hydro-Cy3 for 1 h followed by OD measurement at 530/590 nm. **(C)** Whole cell lysate was prepared from Neo- or Snail-transfected ARCaP cells treated with 1 or 50 μM PMA plus or minus 500 U/ml SOD for 1 h. 100 μl was mixed with HEPES/PBS buffer and 25 μM of Hydro-Cy3 for 1 h followed by OD measurement at 530/590 nm. Results were normalized to protein concentrations that were measured I whole cell lysate using BCA assay. **(D)** 5000 ARCaP-NeoL or ARCaP-Snail med cells were plated in duplicate, overnight in 16-well chamber slides. The following day, cells were incubated with 5 μM MitoSOX reagent in the dark at 37°C for 10 minutes. The cells were then washed three times with warm HBS/CA/Mg buffer. Cells stained with MitoSOX (red) were counter-stained with DAPI (blue) to view the nucleus and images taken with a fluorescence microscope. The results are representative of three independent experiments. Magnification ×40. Data are reported as mean + SD (*p < 0.05; **p < 0.005, compared to PMA).

Hydrogen peroxide ROS-Snail signaling has been implicated with breast cancer [12], while prostate cancer cells have been shown to spontaneously produce hydrogen peroxide [25]. We have previously shown that Snail upregulates superoxide species in ARCaP cells *in vitro* and *in vivo*[13]. We sought to confirm this and elucidate the source of superoxide species in ARCaP prostate cancer cells. We utilized ARCaPE cells transfected stably with Snail that displayed increased Snail expression as compared to Neo control cells (Figure 1A). We treated live ARCaP-Neo and -Snail cells with phorbol myristate-12 13-acetate (PMA) to induce superoxide, and stained with Hydro-Cy3, a novel hydrocyanine (Hydro-Cy) dye, which can detect superoxide and hydroxyl radical and is supposed to be more stable than the DHE stain, and imaged the staining (red). We found that PMA induced superoxide, while less staining was observed when SOD antagonist was added (Figure 1B). More importantly, it appeared as if the ARCaP-Snail cells displayed higher staining for superoxide species as compared to ARCaP-Neo (Figure 1B). These results were also confirmed by using dihydroethidium (DHE) staining which revealed elevated levels of superoxide in ARCaPE cells transfected with Snail (ARCaP-Snail low, -Snail high) *in vitro* as compared to the control (ARCaP-Neo) (Figure 1C). We assayed for the source of increased superoxide species in response to Snail transfection by staining with Mitosox dye that detects mitochondrial superoxide. As shown in Figure 1D, there was a prominent increase in staining in the Snail-transfected ARCaP cells. Therefore, Snail is associated with increased levels of mitochondrial superoxide *in vitro* in ARCaP prostate cancer cells.

### Snail Increases Superoxide levels in LNCaP Cells

**Figure 2:**
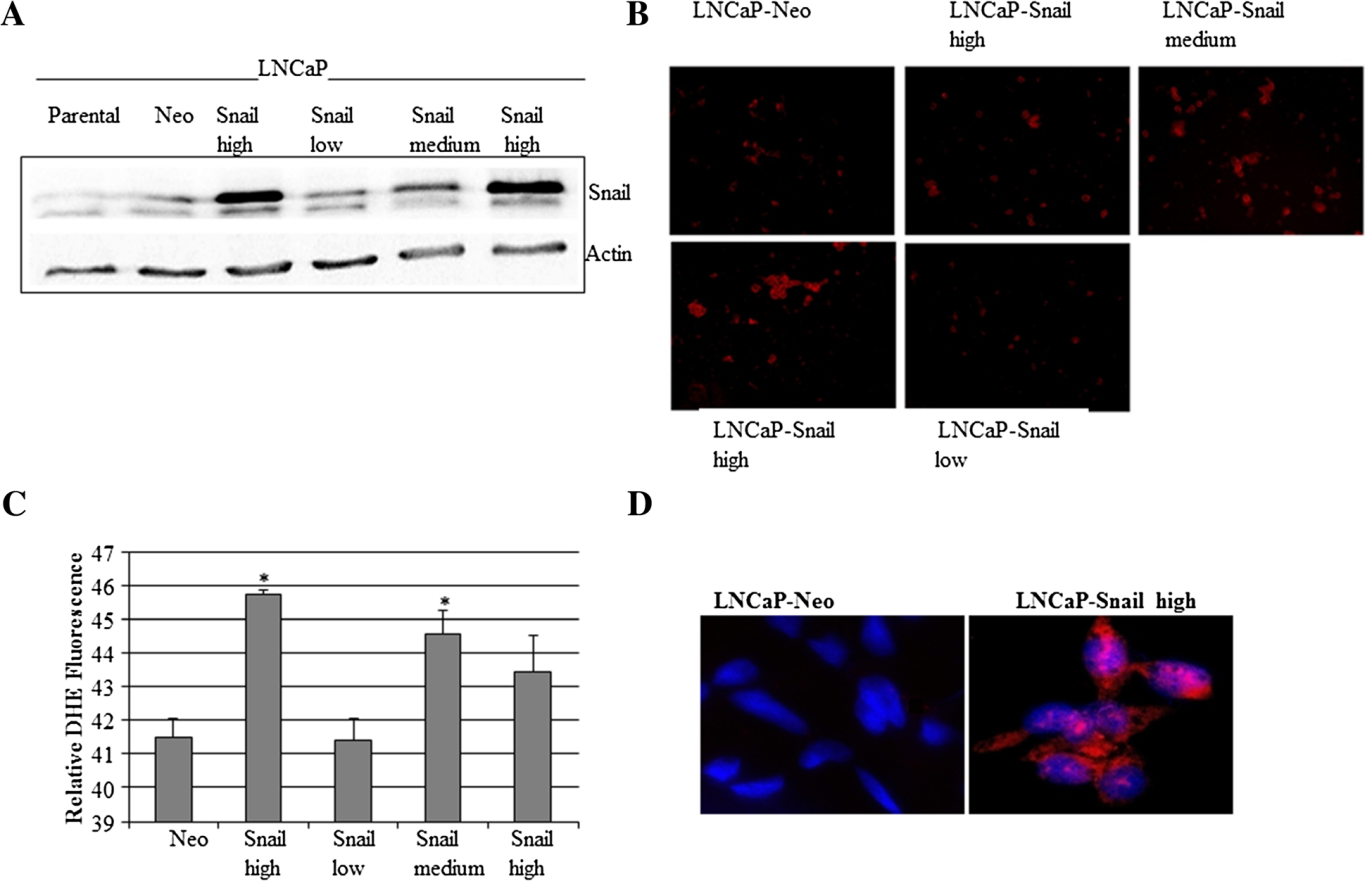
**LNCaP prostate cancer cells stably transfected with Snail display increased superoxide *in vitro*. (A)** Snail expression was tested by Western blot analysis in various LNCaP cell clones stably expressing Snail or empty vector (Neo5), as well as parental LNCaP cells. **(B)** 20,000 LNCaP Neo control or Snail-transfected clones were plated in 6-well plates overnight. Subsequently, the media was replaced with HEPES/PBS buffer containing 25 μM of Hydro-Cy3 for 1 h followed by OD measurement at 530/590 nm. **(C)** Whole cell lysate prepared from Neo- or Snail-transfected ARCaP cells was mixed with HEPES/PBS buffer and 25 μM of Hydro-Cy3 for 1 h followed by OD measurement at 530/590 nm. Results were normalized to protein concentrations that were measured I whole cell lysate using BCA assay. **(D)** 5000 LNCaP-Neo5 or LNCaP-Snail high cells were plated in duplicate, overnight in 16-well chamber slides. The following day, cells were incubated with 5 μM MitoSOX reagent in the dark at 37°C for 10 minutes. The cells were then washed three times with warm HBS/CA/Mg buffer. Cells stained with MitoSOX (red) were counter-stained with DAPI (blue) to view the nucleus and images taken with a fluorescence microscope. The results are representative of three independent experiments. Magnification ×40.

Next we examined whether Snail can increase ROS in an androgen-dependent LNCaP prostate cancer cell line in order to compare the results obtained from Snail overexpression in androgen-independent ARCaP prostate cancer cells. We utilized LNCaP cells transfected with Snail that have previously been shown to undergo EMT [22]. We verified Snail expression by Western blot analysis in the Snail-transfected cells and showed that some clones expressed low (lo), medium (med) and high (hi) Snail expression (Figure 2A). We found that, *in vitro*, superoxide levels increased significantly in the Snail clones that expressed the most Snail (Figure 2B, C). The source of superoxide production in the LNCaP-Snail cells appeared to be mitochondrial in origin, as shown by mitosox staining (Figure 2D). Therefore, our results suggest that in the LNCaP cells, Snail can increase levels of mitochondrial superoxide.

### MSKE and SOD antioxidants decrease superoxide levels in LNCaP and ARCaP cells transfected with snail, and is associated with decreased cell migration

**Figure 3:**
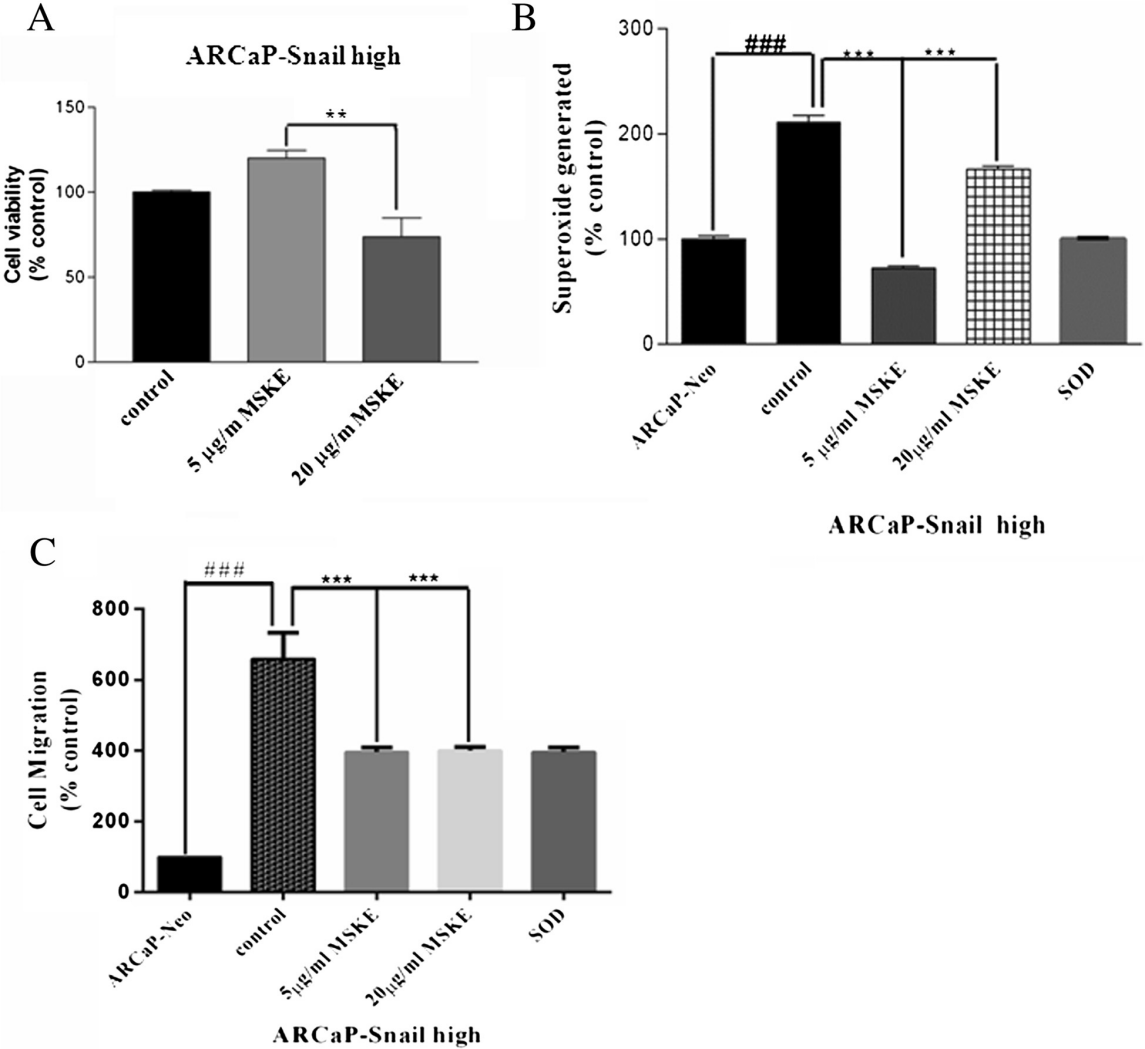
**Muscadine grape extract (MSKE) and SOD abrogates Snail-mediated superoxide species in LNCaP and ARCaP cells and reduces cell migration. (A)** LNCaP-Snail high, ARCaP-Snail med and ARCaP-Snail high cells were plated overnight, serum starved for 3 h followed by treatments with either 5 μg/ml MSKE, 20 μg/ml MSKE, or 500 u/ml SOD for 3 days. Ethanol (EtOH) treated cells Neo- or Snail-transfected cells were utilized as controls. To assay for superoxide levels, whole cell lysates were prepared and 100 μl incubated with 25 μM Hydro-Cy3 for 15 min. OD was measured at 530/590 nm and normalized to protein concentrations. **(B)** LNCaP-Snail high or **(C)** ARCaP-Snail med was treated with 5 μg/ml MSKE, 20 μg/ml MSKE or 500 U/ml SOD for 3 days. Cells were then trypsinized and 50,000 cells plated on collagen-coated boyden chambers overnight. For LNCaP cells, since their cell migratory potential is low, a serum gradient was applied with 0.1% serum in the top chamber and 10% serum in the bottom chamber. For ARCaP cells, the top and bottom chamber both contained 0.1% serum. Cells that had migrated to the underside of the cell insert were solubilized with Sorenson solution and OD assayed at 590 nm to obtain relative cell migration. Results are representative of triplicate experiments performed independently, with data reported as mean ± SD (* p < 0.05; **p < 0.005, compared to EtOH control (-) Snail-transfected cells).

MSKE, a plant product has recently been shown to promote apoptosis of prostate cancer cells, but not normal cells at 20 μg/ml [4]. For studies with MSKE, we decided to focus on the more aggressive ARCaP cells overexpressing Snail. We tested the effect of MSKE on cell viability in ARCaP Snail high cells. As shown in Figure 3A, 5 μg/ml did not affect cell viability after 3 days, while 20 μg/ml MSKE led to a significant decrease in cell viability. We examined the effects of MSKE on superoxide levels in both LNCaP- and ARCaP-Snail transfected cells. Interestingly, we found that 5 μg/ml MSKE was more effective in decreasing superoxide levels when compared to 20 μg/ml MSKE, which was comparable to superoxide dismutase (SOD), a superoxide scavenger (Figure 3B). We further tested whether the MSKE could also affect cell migration. For this we utilized ARCaP-Snail high cells and LNCaP-Snail high cells, which were treated with MSKE for 3 days followed by cell migration assay on collagen type 1 using boyden chambers. As shown in Figure 3C, Snail overexpression resulted in greater migratory potential, which could be abrogated by MSKE in ARCaP and LNCaP cells. Therefore, we show that MSKE can antagonize superoxide production which is biologically associated with decreased cell migration.

### MSKE can revert EMT

**Figure 4:**
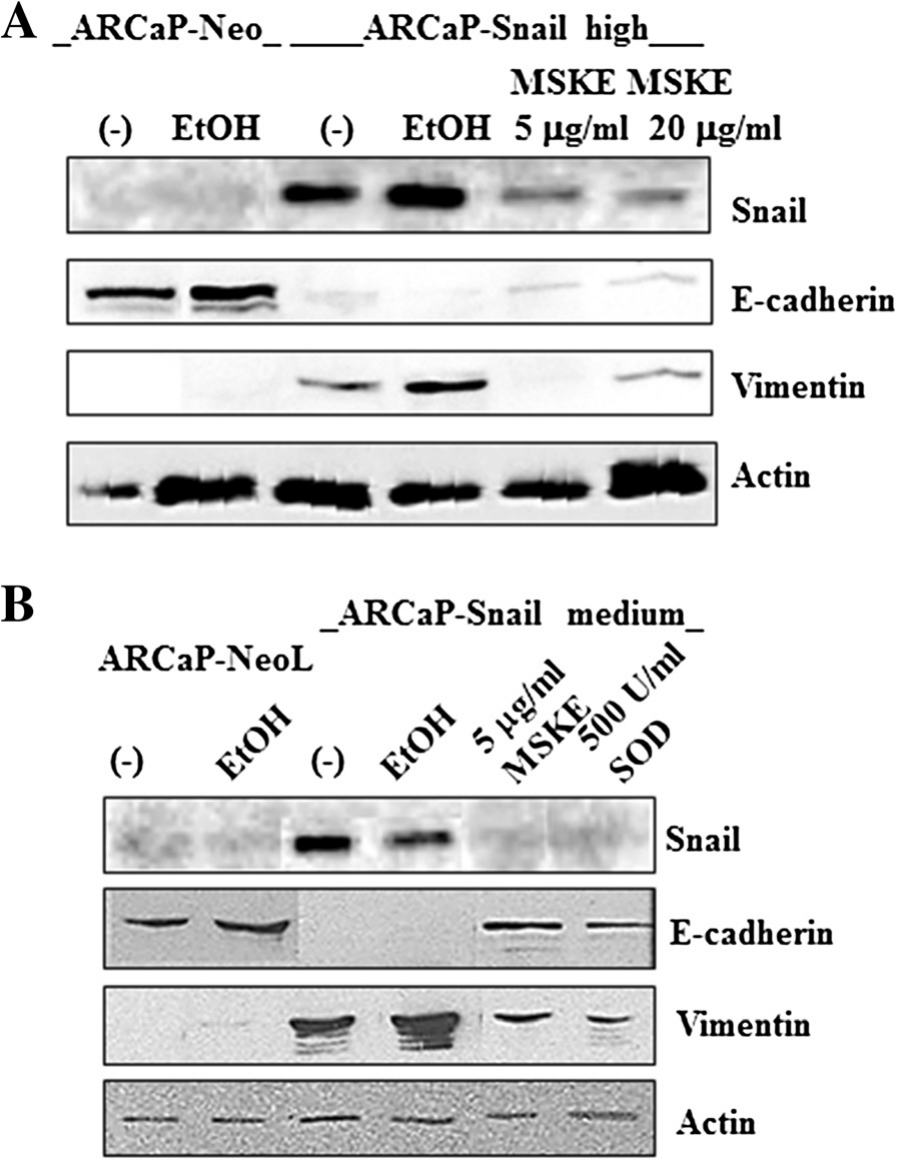
**MSKE and SOD can revert Snail-mediated EMT in ARCaP cells. (A)** ARCaP-Snail high cells were treated with 5 or 20 μg/ml MSKE for 3 days and Snail, E-cadherin and vimentin EMT markers analyzed by Western blot analyses. 5 μg/ml reverted EMT more efficiently than 20 μg/ml as observed by decreased vimentin and increased E-cadherin, as compared to untreated or EtOH-treated Snail high cells. **(B)** ARCaP-Snail med cells were treated with 5 μg/ml MSKE or 500 U/ml SOD for 3 days followed by analysis of Snail, E-cadherin and vimentin by Western blot. Both MSKE and SOD reverted EMT as shown by increased E-cadherin and decreased vimentin. Untreated or EtOH treated cells were utilized as controls. Actin was used as a loading control.

Since Snail can induce EMT and increase superoxide levels, we examined whether there could be a possible link between superoxide species and EMT by using MSKE and SOD antioxidant that can inhibit superoxide. ARCaP-Snail med and –Snail high cells displayed EMT as shown by increased levels of Snail and vimentin, and decreased levels of E-cadherin, as compared to ARCaP-Neo (Figure 4A, B). Treatment of ARCaP-Snail med and-Snail high cells with 5 μg/ml MSKE was more effective at reverting EMT than 20 μg/ml MSKE, as shown by greater re-expression of E-cadherin and decrease in vimentin protein in Western blot analyses when compared to untreated or control EtOH treated cells (Figure 4A, B). Similarly to treatment with 5 μg/ml MSKE, 500 U/ml SOD could also revert EMT (Figure 4B). Both MSKE and SOD could inhibit Snail expression in both ARCaP-Snail clones. These results show for the first time that MSKE and SOD can revert the EMT process in ARCaP prostate cancer cells likely by suppressing Snail-mediated increase in ROS concentration.

### MSKE inhibits STAT-3 activity

Since reactive oxygen species have been shown to activate the JAK/STAT pathway [26] and we have shown that MSKE can inhibit the expression of Snail-mediated superoxide, we wanted to examine the involvement of the JAK/STAT pathway.

**Figure 5:**
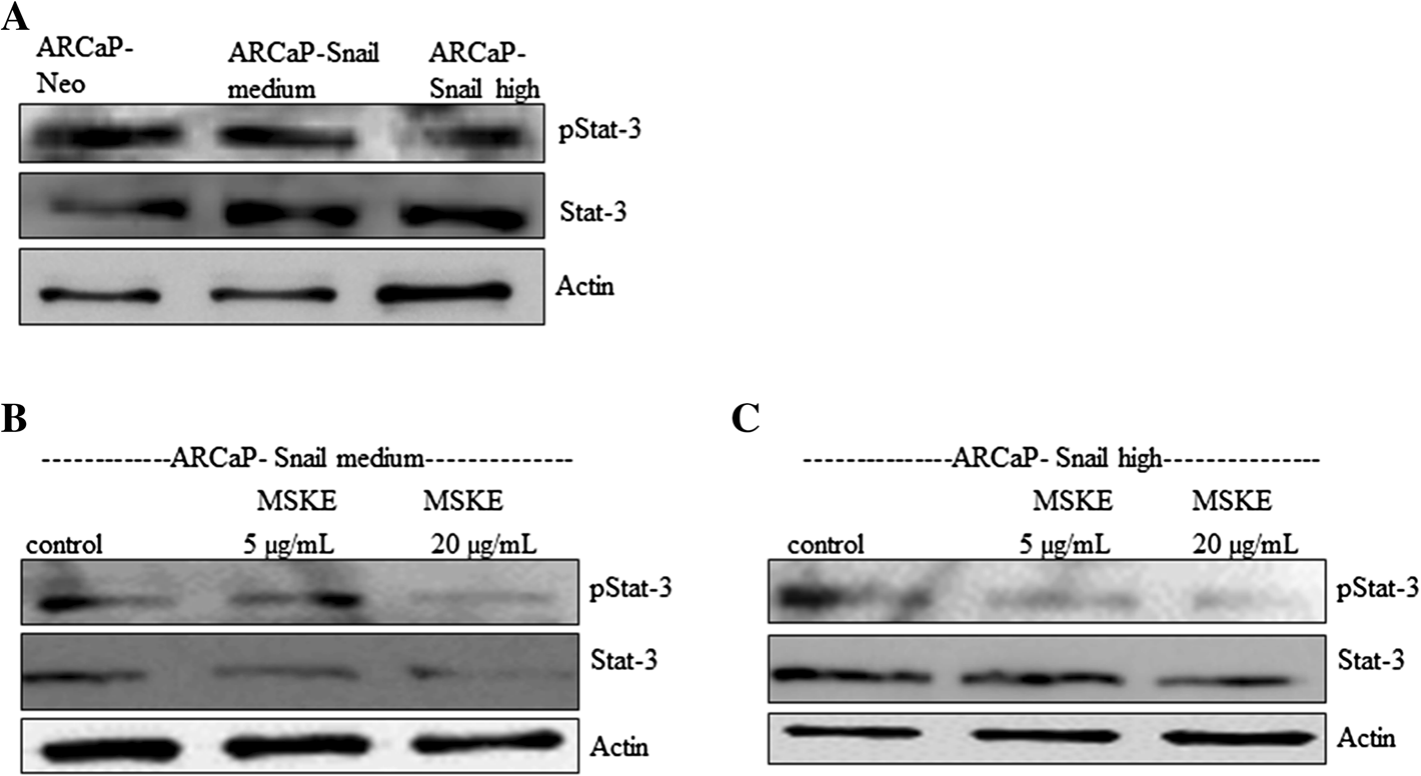
**MSKE may exert its inhibitory effect on EMT and/or superoxide via antagonizing the JAK/STAT pathway in prostate cancer cells (A) STAT-3 and p-STAT-3 levels in ARCaP Neo and ARCaP-Snail med and ARCaP-Snail high (B) ARCaP-Snail med and (C) ARCaP Snail high cells were treated with 5 μg/ml MSKE and 20 μg/ml for 3 days followed by analysis of STAT-3 and p-STAT-3 by western blot.** Compared to untreated or DMSO control treated, 20 μg/ml showed a decrease in STAT-3 and p-STAT-3 as compared to 5 μg/ml MSKE or control. Untreated cells were utilized as controls. Actin was used as a loading control.

ARCaP-Snail med and ARCaP Snail high cells were treated with 5 μg/ml MSKE and 20 μg/ml for 3 days followed by analysis of STAT-3 levels and activity by western blot analysis. Interestingly, STAT-3 activity (p-STAT-3) was inhibited by 5 μg/ml MSKE while 20 μg/ml inhibited both STAT-3 levels and p-STAT-3 (Figure 5). Taken together, our data indicates that MSKE may exert its inhibitory effect in part by antagonizing the JAK/STAT pathway in prostate cancer cells.

## Discussion

This study evaluated the molecular mechanism(s) by which Snail transcription factor may contribute to cancer progression in prostate cancer through regulation of superoxide ROS and whether it can be antagonized by MSKE, a product with antioxidant properties. ROS are important mediators of tumor progression; increased hydrogen peroxide and superoxide species were found in colon carcinoma specimens, while high superoxide levels were present in breast cancer tissue specimens [10]. There has also been a report on increased hydrogen peroxide levels in human prostate tumors [27]. Since ROS is associated with tumor progression, and EMT is also linked to tumor progression [28], additional studies have shown that hydrogen peroxide can mediate EMT [11,29]. Previous studies have shown that Snail can increase hydrogen peroxide and superoxide levels in prostate cancer cells *in vitro* and *in vivo*[13]. However, the role of superoxide in prostate cancer is not well studied and its role in EMT has never been reported. We utilized prostate cancer cells overexpressing Snail, as an EMT model to study the role of superoxide in the EMT process.

Androgen-independent ARCaPE and androgen-dependent LNCaP cells transfected with Snail have been previously shown to undergo EMT [21,22]. In our current study, we have shown that the levels of superoxide increased in both ARCaP and LNCaP cells transfected with Snail *in vitro*. Furthermore, the source of superoxide appears to be mitochondrial in origin, according to the Mitosox staining experiment. Previously, only hydrogen peroxide has been associated with EMT induction in cancer [11,12]. One study showed that transfection of MMP-3 gene into breast cancer cells could induce both hydrogen peroxide and Snail [12].

We tested MSKE, a plant product that has been shown to induce apoptosis and reduce cell proliferation in prostate cancer cells but not normal cells, by antagonizing ERK and PI3K signaling [4]. Muscadine grapes have unique aroma and flavor characteristics. Although a few studies reported high polyphenols content of muscadine grapes, little research has been conducted to evaluate the phenolic compounds bioactivities in any muscadine grape [30]. The major phytochemical constitute of MSKE is anthocyanin 3,5-diglucosides which is different from resveratrol [4]. The phenolic structure of anthocyanins is responsible for their antioxidant activity (ability to scavenge ROS) [5]. In our study 5 μg/ml MSKE led to a mesenchymal epithelial transition (MET) characterized by reexpression of E-cadherin and reduced vimentin levels. However, the higher dose (20 μg/ml) that has been shown to induce apoptosis in LNCaP cells [4] and decreased cell viability in ARCaP Snail high cells, was not as effective at inducing MET and affected reexpression of E-cadherin, but not vimentin levels. Further support came from examination of superoxide levels showing that 5 μg/ml effectively inhibited superoxide levels in ARCaP-Snail cells by more than 50%, while 20 μg/ml MSKE had minimal effect. This suggests lower doses of MSKE can be more effective at reverting EMT than higher doses, possibly by inhibiting superoxide. This is further supported by the data showing that the superoxide inhibitor, SOD, could significantly inhibit superoxide accompanied by a MET. This would be the first report of regulation of EMT by superoxide species. Interestingly, both doses of MSKE as well as SOD could inhibit Snail expression as well as cell migration. We do not currently understand why 20 μg/ml MSKE is just as effective as 5 μg/ml MSKE at reducing Snail levels and cell migration, yet it does not revert EMT or reduce superoxide levels as effectively. Studies have shown that it is possible that STAT-3 activation might be induced by ROS generation [26]. In our study we observed that while 5 μg/ml MSKE affected STAT-3 activity but not levels, 20 μg/ml MSKE did show an inhibition of STAT-3 levels and STAT-3 activity. There is obviously a difference in signaling between the low and high doses of MSKE and the high dose may act by inhibiting the JAK/STAT pathway leading to apoptosis while the lower dose may also inhibit STAT-3 activity and inhibit EMT. However, both doses inhibit Snail expression. Snail has not only been implicated in EMT but also in cell survival [31]. The difference between low and high dose of MSKE needs to be investigated further.

## Conclusions

**Figure 6:**
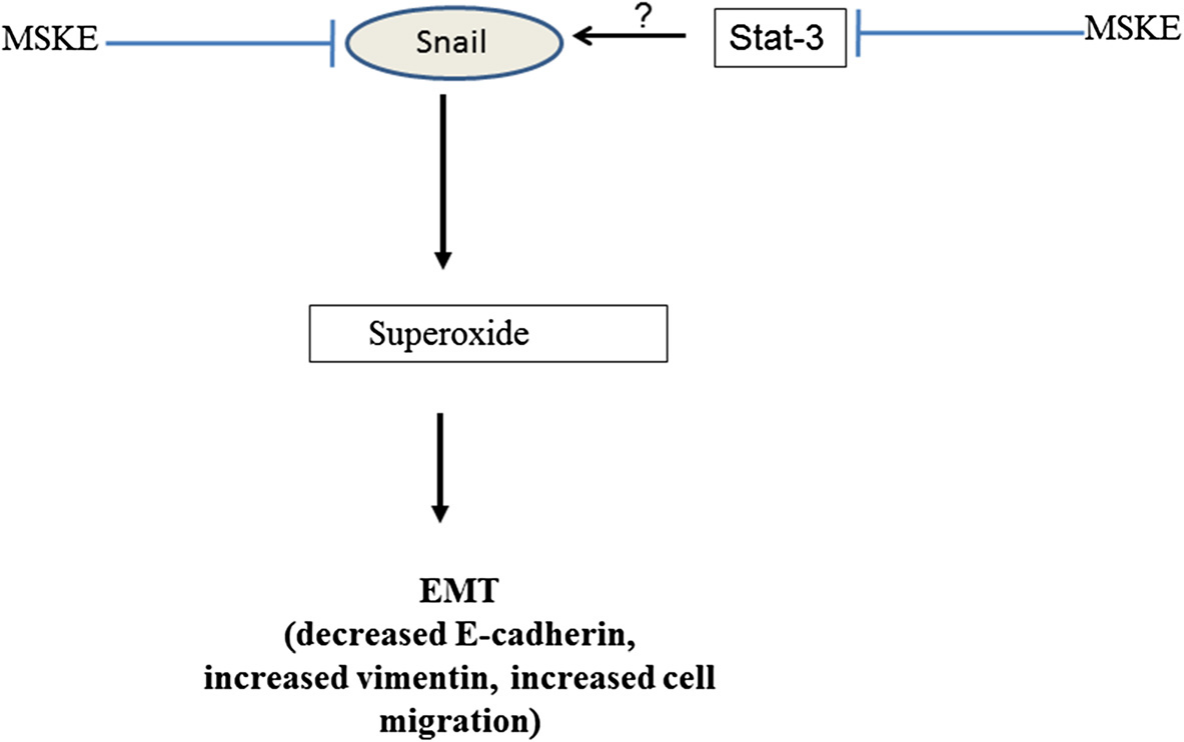
**Pathway by which MSKE antagonizes Snail-mediated EMT in prostate cancer cells.** Snail induces EMT via superoxide production which is characterized by decreased E-cadherin expression, increased vimentin expression and increased cell migration. MSKE antagonizes Snail-mediated EMT by inhibiting Snail expression possibly via inhibition of Stat-3 activity and superoxide production.

Collectively, our results indicate that Snail leads to increased levels of mitochondrial superoxide and EMT (Figure 6). Furthermore, MSKE and SOD reverts EMT by targeting Snail expression (Figure 6), underscoring the importance of targeting these pathways with various inhibitors and antioxidants. These studies show that superoxide species may play a role in the EMT process and that use of various antioxidants such as MSKE may be able to antagonize EMT and prostate cancer progression in future.

## Abbreviations

MSKE: Muscadine grape skin extract; EMT: Epithelial mesenchymal transition; ROS: Reactive oxygen species; STAT-3: Signal transducer and activator of transcription 3.

## Competing interests

The authors declare that they have no competing interest.

## Authors’ contributions

LB: performed experiments, analyzed data and generated figures and manuscript. PB: performed experiments, analyzed data and generated figures and manuscript. RSA: conducted DHE studies. KK: synthesized and provided the HydroCy3 dye. NM: synthesized and provided the HydroCy3 dye. VOM: designed project, project coordination, and manuscript preparation. All authors have read and approved the final version of the manuscript.

## Pre-publication history

The pre-publication history for this paper can be accessed here:

http://www.biomedcentral.com/1472-6882/14/97/prepub
